# Distrust, trauma, doubt, and protective reactions to coronavirus disease 2019: cautionary tales and lessons to learn for future pandemics: a case report

**DOI:** 10.1186/s13256-025-05162-w

**Published:** 2025-03-21

**Authors:** Jacinda K. Dariotis, Dana A. Eldreth, Stephanie M. Sloane, Iffat Noor, Rebecca Lee Smith

**Affiliations:** 1 Department of Human Development and Family Studies, The Family Resiliency Center, College of ACES, The University of Illinois at Urbana-Champaign, 904 W Nevada St., Urbana, IL 61801 USA; 2 Department of Biomedical and Translational Sciences, Carle Illinois College of Medicine, The University of Illinois at Urbana-Champaign, 506 S Mathews Ave., Urbana, IL 61801 USA; 3 Carl R. Woese Institute for Genomic Biology, The University of Illinois at Urbana-Champaign, 1206 W. Gregory Dr., Urbana, IL 61801 USA; 4 Department of Kinesiology and Community Health, College of Applied Health Sciences, The University of Illinois at Urbana-Champaign, 1206 S. Fourth St, Champaign, IL 61820 USA; 5 Department of Pathobiology, College of Veterinary Medicine, The University of Illinois at Urbana-Champaign, VM BSB 2418, Urbana, IL 61801 USA

**Keywords:** COVID-19, Vaccine decisions, Distrust, Prevention, Public health messaging

## Abstract

**Background:**

Vaccine uptake has declined since the coronavirus disease 2019 pandemic began. The pandemic changed people’s perception about vaccination due to factors such as increasing mistrust in government, spread of misinformation, fear of side effects, unclear communication, concerns about rushed vaccine development, and opposition to mandates infringing on personal choice. Understanding different perspectives on vaccine decision-making is crucial for informing effective approaches to communicating about vaccines.

**Case presentation:**

This study presents three cases with varying attitudes and behaviors about vaccination for coronavirus disease 2019, traditional childhood illnesses, and influenza influenced by different contexts and experiences. The cases span the continuum of vaccine hesitancy and uptake, from distrustful and resistant (Alexis, 56-year-old non-Hispanic White American female), through resentment for mandated uptake of the expedited coronavirus disease 2019 vaccine versus trust of long-standing preventive vaccines (Nia, 51-year-old non-Hispanic Black American female), to accepting and adopting (David, 38-year-old non-Hispanic White American male). These cases have similarities and differences across ten key “themes,” including vaccine attitudes; decision-making motivations; prioritizing family’s health; influence of past vaccination trauma on decision-making; significance of social support; the importance of information to guide decisions; (dis)trust in news, social media, and politicians; disappointment in humanity; future recommendations including respecting individual autonomy and providing the necessary information for individual decision-making; and openness to future vaccines.

**Conclusion:**

The long-term impact of the public health response—including vaccine mandates—and aftermath of stigmatization of people with differing and less socially desirable vaccine beliefs on vaccine uptake and health and medical service engagement remains unknown. By drawing on rich, nuanced information collected from individuals at a time of intense national dialogue around vaccines, these three case studies offer unique and novel insights into how the dialogue around vaccine uptake should evolve to meet the needs of different people. These findings have implications for broadly promoting public health engagement by hearing varied experiences and tailoring approaches to reach diverse groups of individuals. Findings from these cases provide insights and recommendations for tailoring future pandemic-related responses to audiences with similar beliefs and experiences as those presented in these cases.

## Background

People who choose to remain unvaccinated may be motivated by different rewards, fears, and values than people who express confidence in vaccines, and experience barriers (for example, access to quality education, healthcare resources) that undergird personalized risk–reward calculations driving behavior. Combined with medical distrust, conspiracy beliefs, and mis/disinformation being at all-time highs [1–3], coronavirus disease 2019 (COVID-19) vaccine uptake has been incredibly low at < 69% for the full vaccine and < 15.9% for the bivalent booster among eligible Americans [4, 5]. As of July 2024, COVID-19 vaccination rates among adults aged 18 years or older remain low at 22.6% [6]. The COVID-19 pandemic has changed people’s perception about vaccination due to increasing mistrust in government, spread of misinformation via social media, fear of side effects, and lack of clear instructions about the vaccine from the Centers for Disease Control and Prevention (CDC) [7]. Other factors reducing vaccine acceptance include fears about the rushed development of the COVID-19 vaccine and the unknown duration of immunity [8]. In fact, from January 2020 to August 2020, acceptance of COVID-19 vaccines decreased from 70% to < 50% [9].

In adults, the intent to vaccinate against COVID-19 was driven by mortality rates [10], perceived risk of infection [8], and disease severity [11]. A scoping review of articles published during the COVID-19 pandemic found that intent to vaccinate against COVID-19 in adults was influenced by demographic factors (for example, race, gender, education level), social factors, vaccination beliefs and attitudes, vaccine safety and effectiveness, influenza vaccination history, and infection prevention [12].

In a study of US mothers, Walker *et al*. [13] found that mothers’ intent to vaccinate their children was not solely predicated on prior vaccine attitudes or behavior, because health beliefs are affected by contextual factors related to the vaccine and severity of the disease. They also suggested that astute health decisions are contingent on accurate perceptions of the cost–benefit; however, emotions such as the feeling of being threatened may override rational decision-making based on accurate information. Healthcare and social workers caring for vulnerable population have additional considerations. A Swiss study of these workers found that the decision-making process for vaccination could not fit neatly within categories of pro- or anti-vaccine [14]. Primary patterns identified as driving the decision-making process included principle-driven, the tradition-driven, the emotion-driven, and the reflexive. These patterns are influenced by personal versus collective relevance assessments. These workers also had to navigate challenges such as the infodemic, depersonalization, moralization of the vaccination decision, and fear of workplace discrimination. Moreover, decisions for healthcare workers are often rooted in fiduciary duty and ethical responsibilities [15].

To understand the motivations for one’s decisions, it is important to understand their lived experiences. Previous studies have not investigated in sufficient depth past experiences and attitudinal and contextual motivations for or against vaccination. Here we showcase three case studies, each representing different types of decision-makers, with grounding in their past experiences, current beliefs about COVID-19, and intended behaviors during future pandemics. We highlight their commonalities and differences and propose recommended approaches to vaccine promotion for public health and medical professions moving forward that respect the lived experiences of different types of decision-makers.

## Methods

### Study design

The primary aim of this case report was to explore perspectives and decision-making processes regarding vaccine uptake of the COVID-19 vaccine and other vaccines. The cases used in this report were adults who participated in a larger mixed methods COVID-19 study of 506 parents and childcare providers. The primary aim of the parent study was to investigate the lived experiences of parents and childcare providers during the pandemic and adherence to public health guidelines, including vaccine uptake. The study was approved by the university institutional review board. Participants provided informed consent through an online eligibility screener. Inclusion criteria for the parent study included parents or caregivers aged 18 years or older who were currently caring for minor children or who were childcare providers, and participants had to be proficient in written and spoken English and located in the USA. A subsample of 45 participants were selected for semi-structured interviews using a purposive sampling strategy on the basis of survey responses to ensure vaccine attitudes were captures across a continua of pro- to opposed attitudes. Participants who completed the interview received a $25 e-gift card.

### Case inclusion

The three cases in this report were selected on the basis of (1) the richness of their data regarding their decision-making processes for vaccine uptake, opt-out, or hesitancy during and post-pandemic and (2) diverse experiences influencing their perspectives and behaviors. Pseudonyms are used for each case to protect participant identities. Case 1, Alexis, was selected because she was engaged in the medical field as a nurse, had a child with disabilities, and experienced past trauma with vaccines resulting in her decision not to vaccinate herself or her child against COVID-19. Alexis left her nursing position soon after the pandemic began because she disagreed with how it was being managed by healthcare. Case 2, David, was selected because he was employed as a risk assessor who made decisions on vaccination on the basis of research and statistics. He had four children, one of whom had preexisting conditions that heightened his caution of exposing his family to COVID-related risk. Case 3, Nia, was chosen because as a childcare provider, she reported being forced to take the COVID-19 vaccine due to her employment without having enough time to research and make informed decisions. She experienced symptoms after receiving the COVID-19 vaccine that she attributed to the vaccine. Despite previously being pro-vaccines, her experiences of being mandated to vaccinate and then suffering perceived long-term negative health side effects of the vaccine resulted in her being reluctant to receive future vaccines and to be highly skeptical of vaccines, media, and other influences.

### Procedures

Open-ended, semi-structured interviews were conducted via Zoom or in-person on the basis of the preference of the participant. The interviewers asked questions regarding COVID-19 lived experiences, vaccination status, views on vaccines, coping and adaptations made, resources needed, recommendations for public health policy, and messaging in future public health crises. The interview duration was as follows 149 minutes (Alexis), 110 minutes (David), and 113 minutes (Nia).

## Results

### Case 1: Alexis

*Case History*. The first participant case we present is that of Alexis. She identified as a 56-year-old divorced White female with one 15-year-old son with special needs and two aged parents. She was a nurse, both in the emergency room and then at an older age day care facility. Alexis had experienced COVID-19, did not receive the COVID-19 vaccine, and strongly disagreed when asked if she intended to vaccinate herself or her son against COVID-19 in the future. She believes in a God and places strong reliance on her “beautiful innate immune system I came into this world with at birth!” She began self-health improvements—healthier diet, more exercise—approximately 3–4 years before the pandemic (5–6 years before our interview).

*Pandemic Related Re-Traumatization*. When the pandemic began, she had been employed as an emergency room (ER) nurse for 14 years. Within the first month of the pandemic, she chose to quit her job for two reasons. First, she “could see that this [the pandemic] was not being handled appropriately.” Second, she needed to care for her son who was sent home to be home schooled. In her words,

*I walked out of the hospital and never returned. So people thought I had absolutely lost my mind. It was at the first job that I had literally walked away from. I was there almost 14 years. And with the meditation, with my morning prayers, with everything, I knew that I was making the right decision… I have a sticky notes all over my kitchen and one of them says if we don’t have peace with something then don’t do it. And I totally had peace with walking away from that*.

Alexis recalled that although many ER health professionals are not fazed by much (“those people who work in the ER are not really frightened of anything and it’s just our nature. We kind of thrive on adrenaline”), she had real fears about her son becoming sick because he had been previously hospitalized for respiratory illness. She was traumatized by her son’s negative response to the measles, mumps, and rubella (MMR) vaccine at age 3, which she admitted postponing until she was forced to do for him to go to school.

After her son’s MMR experience, Alexis vowed to never put other vaccines in her or his body. As she described, “I’m the nurse that would, that fought tooth and nail to not get flu shots. So this will never be in me. It will never be in my son.” She reported historically being mandated to receive the flu shot as a condition of her employment for about 2 years.

*Pretty much that I was forced to get it [flu shot]. Maybe 2 years as a mandate, which I do not agree with. That is my body and my choice. And I shouldn’t have to be forced to do that. And I would literally write on the form they wanted me to sign that I was signing under. I wouldn’t sign my name. I just wrote on the line “under protest.” And, I would write all around the edges “if I get Gillain-Barre, you will take care of me 24/7, and my son with disabilities 24/7” and on and on*.

Flu vaccine mandates further fueled Alexis’ distrust in vaccines when her son became ill in 2016. Alexis went on to say that her son was hospitalized for a week within 2 weeks of her receiving the flu shot and had to receive respiratory treatment for an additional 3 months. She attributed his sickness to her flu shot and never received one again: “I've finally got a physician to sign a medical exemption for me so that I didn’t have to do it [get the flu shot] again.” Both because of her personal experience with her son’s illnesses and what she knew from flu vaccine effectiveness, she had no trust in it. Further, she believed vaccines “are doing something to your immune system that God gave you that’s perfect. So I don’t think we should interfere with that.”

Alexis explained that since her son’s negative MMR reaction at age 3 years, he always receives vaccination exemptions, “a religious exemption was the only way that they would let that happen at the school district.” Her son’s pediatrician advised her not to have her son vaccinated against COVID-19. She recounted that a cousin’s 15-year-old athletic son had major cardiac complications after receiving the COVID-19 vaccine shot and now has a pacemaker. These sequential experiences reinforced her distrust and doubts related to vaccines.

She prioritizes her own and her son’s health and values the immune system response without vaccines. Alexis was an educated health professional who took a vested interest in knowing what is in medicines. As she explained, “I have done my research since my son was little about all the evils and all the like additives, and the having metals and the aluminum and all the s*** that gets in there. That's not what needs to be injected into people.” Alexis did admit to a recent exception she made when she became vaccinated for Tetanus-D because it was required for her and her son to participate in a big hiking trip for Scouts in 2021. She reluctantly agreed and insisted she receive it in a way that put the least amount into her body. With respect to COVID-19, she adamantly believed that mRNA is not a vaccine (see below).

*Renewed Commitments—Religion, Prioritizing Family and Health*. During the pandemic, Alexis had a renewed commitment to religion. She began attending church more. She connected with a spiritual coach and went on a spiritual journey across the country to spend time in a retreat. She found her “love tribe” during the pandemic, which was an online group that went through a spiritual workgroup together each week and supported each other through a course of miracles. At the time of the interview, Alexis’ love tribe continued to meet monthly online to check in with each other. As Alexis described,

*The expansion of a worldwide group of love tribe was something that I wouldn’t have experienced without what was going on in the rest of the world because I slowed down. I had the time, and I was reaching out, in all kinds of spiritual ways to figure out myself and what was coming next*.

Other changes she made included home schooling her son even though he could have returned to school. She also had her son in numerous activities such as Scouts, taekwondo, and hockey. Although she had less financial stability, it concerned her parents more than it did her. Her renewed faith in a higher power was liberating and revealed to her the many fortunes she had. As Alexis described,

*Financially, in general, it’s not as plentiful. If that’s what you what you want to say. But it also opened our eyes to all the ways that you really are blessed and really are prosperous. That it doesn’t come necessarily in the form of money. That there’s many other blessings. And what’s really cool is when you give up that control to that higher power, whatever that is for you. And you allow those things in. It’s amazing how you get what you need. It may not be what you want, [laughs] but it is what you need*.

The pandemic helped with prioritizing what people spend money on, keeping each other safe, and spending time with family. She kept her son from playing hockey for a year to keep him safe. Given her nursing background, she knew the importance of wearing masks. Before leaving her hospital job, she took many N95 masks that had been fitted to her. She anticipated they would be needed. Alexis wore masks until she thought they were no longer needed or in contexts she did not think they were healthy to wear (like outside). However, she respected people’s desire to wear masks longer or outside if that makes them comfortable, noting she would not tell them to do otherwise.

*Significance of Social Support, Gratitude, and Forgiveness*. When asked about the most important silver lining, Alexis mentioned her love tribe. The need for and importance of social support was very strong and she found a network across the globe, which she referred to as her love tribe. She espoused the belief that “there’s only two ways to live; it’s in love or it’s in fear.” This social network helped her throughout the pandemic, providing support, recommendations, and connection. As she explained, “the need for mental health, you know assistance for people, especially with the isolation. Thankfully I did get that love tribe, and I did reach out and do those different things.” During the pandemic she also developed a deeper sense of gratitude. She kept a gratitude book where she would write five things every day for which she was grateful. In her own words, she described the book and the evolution her entries as follows:

*[A] book that I write like five things every day. Actually, it was really interesting. It went from things like when I was working at the hospital, from things like, I’m grateful for a hot shower or chicken to things like the trees are budding today and there’s a rabbit here [laughs]. I mean, it was like just this whole like different, all of gratitude*.

Alexis took seriously her commitment to live in love. She did so by empathizing with others and accepting that they make choices on the basis of their contexts and constraints. She acknowledges that some approaches taken during the pandemic were based on fear and were forced. To move forward, heal, and evolve as people, she recommends forgiveness be practiced by all. She defined forgiveness as “see[ing] through the layers of muck and mire to that love and light that we all were given.” Experiences with her love tribe and gratitude practice were part of the journey that brought her to the conclusion that forgiveness was needed. She describes her journey to forgiveness this way:

*I’ve kind of gone almost to the extreme… in spiritual way, in a loving way. We’ve all made choices. We’ve all been buffaloed by certain things. There’s been a lot of propaganda and a lot of fearmongering and coercing. And so, yeah, a whole lot of forgiveness needs to happen on all angles of this, so that we can push our way forward, and admit what we can do to make people better*.

*Scientifically Distrusting of mRNA: Need for Informed Consent*. Alexis adamantly believed that mRNA is not a vaccine. As she described,

*What is frightening is that mRNA technology is not a vaccine. And that’s just scientifically period. They [the CDC] change the definition of a vaccine to fit that what they wanted. The CDC is evil. In that regard they plan from my own understanding, to put this technology into damn near every shot. Now in the future, and so I don’t plan to ever have anything past my last TD [tetanus and diphtheria]*.

She is concerned about the future of any vaccine because she contends mRNA technology will be used for all vaccines in development: “that they want to use mRNA technology in everything. And I think that to go in and mess with your genetics on that level is not smart.” As a reminder, Alexis has a nursing degree and practiced in the ER for nearly 15 years. She is concerned about mRNA and credibly communicates that to others even if all of her facts may not be accurate. As she eloquently explained in the interview:

*But to actually inject things that cross your blood-brain barrier. No matter what they are, is not a safe thing to do for any of humanity that includes all the animals. And, all they want to vaccinate our animals with this stuff and all the food we’re going to eat with all the cattle and everything… So, my son and I just this week have been studying in the biology book how that DNA gets to the messenger RNA and gets transcribed to make the amino acids and the proteins kind of like, I mean, even in a high school science book, you can tell that that’s not where you want something to go wrong. That’s not where you want someone to be messing. So they’re not vaccines. They’re gene modifying injections*.

When asked about what is needed in the future, she said better informed consent so that people understand the long-term implications of vaccines in terms of their health, financial well-being, and functioning. As she warned.

*B[y] signing off on that, you basically give away everything. There will not be any way you can go back and get money from that pharmaceutical company, from that person injecting you from that hospital system, from the doctor’s office. They are all completely indemnified from any kind of problem that you have*.

Alexis made several recommendations for future communication and what could have been done differently with the COVID-19 pandemic. First, medical and public health communication needs to provide more detailed and digestible information about what mRNA actually entails and what vaccines developed with it do to one’s body. Second, “Most importantly is that we all need to be treated like individuals. We’re not herds of cattle to go in and all get the same thing. Does that make sense? I mean as to mandate specific things or even like with the mask.” She went on to explain that her mother had chronic obstructive pulmonary disease (COPD) and could not walk far without major difficulties breathing. Mask wearing was detrimental because she had to breathe in her own breath. “So one size does not fit all. I think that would be my biggest message, and that goes true for children as well.” She went on to say that because the risk of death for children was so small, forcing them to be vaccinated was “pull out a big word here, genocide. That we’re doing to our children by putting this in them because their risk was like 0.0003% that this would have a bad outcome. Yeah.”

*Stigmatized for Being Different*. Alexis experienced not only trauma from her son’s negative medical experiences to vaccines, but also from being made fun of because her vaccination beliefs were different from others. She was trying to keep her son safe and his special needs meant holding varying attitudes and behaving in different cautionary ways. She described:

*I just think that it all needs to be looked at more from an individual standpoint and not from, yeah not from a political thing. They made it fun to judge people that were different, and I guess that’s near and dear to me. Having a child with special needs, you know, it’s like No. We've forgotten how to honor the individual. The herd mentality is scary*.

Alexis, her son, and their closest friends were ostracized, coerced, and peer pressured because of their beliefs not to be vaccinated. This was very impactful for them.

Alexis noted that she did not trust any media outlets because they were owned by “Big Pharma” and that many congressional representatives receive campaign funding from Big Pharma. She went on to recount how she had recently looked up a fact from the 1960s and the Wikipedia post had been updated only 5 days prior, questioning why historical facts need to be updated when “that should be solid.” “What’s the truth?” she asked not only of media depictions, but of the vaccine.

When asked how public health professionals could reach her and others who share her perspective and what should their messaging be, she spoke about protecting people’s right to keep their vaccine attitudes and behaviors private. “That your health is a private conversation protected by the law with your doctor. I don’t think we should be talking about any of it out here, anyway… I think that that that we have done what has happened in the last few years is that a huge disservice to the medical community in general… it has destroyed a lot of that confidentiality between a doctor and a patient.”

Her biggest take-away from the pandemic was “focusing on gratitude and abundance, gives me clarity of choices and solutions. So I guess the my biggest takeaway, maybe is that I have Strengthened my resolve with my higher power, that gives me the strength to Lock in that trust And not fear.” She ended the conversation with this saying that she placed in her room since May 2020: “Universe, put me in the places you want me to be with, the people you want me to be with, doing the things you want me to do. Thank you for all the joys and the challenges of my life.”

### Case 2: David

*Case History*. The second participant case is David, a 38-year-old married White male with four children, one of whom had health concerns. At the time of the interview he was fully vaccinated and boosted and had contracted COVID-19. David had a graduate degree and worked in risk management. He used data, science, and risk tolerance to guide his family’s decision-making around vaccination. He was a proponent of vaccines in general, but reviewed literature and data related to new vaccines before making uptake decisions.

*Privy to More Information about the Pandemic*. He was a risk management specialist and had worked with agencies such as Federal Emergency Management Agency (FEMA), which gave him a deeper understanding of the severity and longevity of the COVID-19 pandemic from the beginning. As he explained,

*So professionally I’m in risk management…Pretty much immediately at the beginning of the pandemic… you’re having meetings with other parent organizations… “maybe we need to hold off on activities four or six weeks until this dies down.” And the information I’m getting in the background from [agency] is “we’re canceling everything for the next eight months.” It’s not okay*.

David struggled with the disconnect between the level of information he and his family were privy to due to his job and the information available to the general public. David’s knowledge of the guidelines for vaccination was sometimes ahead of the doctors’, so he had to advocate for his youngest son.

*So a lot of times related to vaccines like we had gone through all of like the administration guides of how the vaccines could be administered to our children and….What the precautions and what the special cases were, and so like for our 5 year old who was at higher risk. Like we were aware that he could be vaccinated well in advance of his doctor providing us with that option. So we had to go through and kind of explain. Why what the ACIP [Advisory Committee on Immunization Practices] had just approved applied to him and he was eligible to come in and get vaccinated even though he hadn’t turned 5 yet.*

*Prioritizing Safety of Children*. David had a child with health conditions that placed him at higher risk for contracting COVID-19, which dictated the actions of the entire family. David and his family took the pandemic seriously and were “cautious of hygiene and illness prior to the pandemic.” During the pandemic, he and his family took many precautions to reduce their risk of exposure. All four of David’s children participated in remote learning until spring 2022, staying out of school longer than most children. His family avoided contact with others for an extended period of time and stopped all extra-curricular activities. In his words,

*We weren’t going out to a lot of public places. So we were kind of across the board intentionally isolating so doing curbside pickup for anything we needed coming into our household. All of our activities we were pretty much doing on our own, spent a lot of time at the local forest preserves doing tons of hiking and outdoor activities with the kids and biking and walking around and generally just kind of keeping our distance from other people*.

They occasionally interacted with one family across the street but only outside and always masked.

*Importance of data and science for making informed decisions*. David and his family used data and science to guide their actions related to COVID-19. He described them as “pro-vaccine before the pandemic” and talked about how they occasionally reviewed literature on specific vaccines or additives. All members of David’s family received the standard childhood vaccines, the annual flu vaccine, and children of eligible age have received the human papillomavirus (HPV) vaccine. During the pandemic, they began to review data about vaccines more closely as described by David:

*I would say during the course of the pandemic we definitely started looking at that [vaccine data] much more closely. So looking at the ACIP and the data as it came out; trying to stay abreast of the information that the people approving those vaccines were looking at themselves so that we could feel comfortable with the decisions moving forward. And I think looking at the data and we didn’t have concerns about kind of the speed at which the vaccines were rolled out, but the technology had been really building for years when they’re looking at MERS and SARS. And this wasn’t as novel as kind of the mainstream media seem to be picking up on sometime. So we were definitely proponents for it*.

The family all received COVID vaccines and boosters as they were approved.

David is open to future vaccines for himself and his family and would use the same cost/benefit analysis to make a decision about uptake.

*Yeah, I would certainly consider it. Just like with all the other shots, we would look at the data and efficacy and health risks and look at that risk benefit ratio and try to see what our perceived risk and of exposure and health risks of getting that shot and the likelihood that it would prevent serious outcomes*.

Minimizing risk guided David’s decision-making around vaccines and his family’s health and safety in general.

*View of Society and Future Recommendations*. During the course of the pandemic, David developed “a much more pessimistic view of the society in which we live both in people’s understanding of basic scientific information that’s presented to them.” However, he used this realization as motivation to help educate and persuade those around him to get vaccinated and to comply with masking and distancing recommendations.

*The role we played was kind of this secondary source of information for people who were sort of either opposed to a certain mitigation strategy or were indecisive. Someone would reach out to us, we would try to kind of tailor the information and response back. Give them the information that either directly disproved their point using data or gave them the resources to explore it themselves*.

Overall, the pandemic and isolation did not have a major negative impact on David or his family. They did not experience economic stress and felt confident in their ability to access and digest information and make rational decisions to keep their family safe.

David’s biggest frustration was that, as a society, we seemed to learn nothing from past pandemics. He viewed the political and nationalistic themes between the 1918 and COVID-19 pandemics as very similar. Although a focus on medical, health, and public health aspects is important during a pandemic, David cautioned that it is not enough. Rather, the social and political implications it has for people’s lives need greater consideration and prioritization. For future pandemics he explained,

*I would really hope that we can collectively study not just the health response and what strategies work best, masking or hand washing or whatever is applicable to the transmission method for the next pandemic or epidemic. But also, how that translates into a lot of the social aspects as well. So how does that impact other aspects of people’s lives, from education and health and how that might shape politics and things like that. So, I would hope there could be more thought to things like that*.

David also remarked that inconsistent messaging about masking early on in the pandemic undermined the public’s trust for the remainder of the pandemic.

*I mean certainly the initial reluctance to promote masking externally. It makes sense policy-wise because there was a shortage of masks and trying to preserve it and get them to first responders and avoid hoarding and things like that. But setting that messaging seemingly intentionally knowing that masking would have been effective from the beginning in order to preserve those, I feel like they lost the opportunity to start that messaging early on. And so you had this switch from no one needs a mask to now suddenly you need a mask again*.

For future pandemics, David suggests presenting information in an objective way and emphasizing the economic impact of getting seriously ill as well as the impact it will have for caring for loved ones. Implicit in his recommendations for how public health professionals should communicate with the public is the need to provide the best information for them to make an informed decision about meeting their needs and goals rather than stating mandates. To public health officials he suggested:

*You are not there to take something away from them. You are not there to dictate what they have to do. You are trying to provide them information so that they can make their own decision… who is going to be caring for your children and providing for them while you're out of work recovering? Yes, you may have long-term health coverage, but will you still be earning wages while you’re recovering from this for several months?*

If framed appropriately, people will know how their decisions will impact how well they meet their family caregiving and economic responsibilities.

### Case 3: Nia

*Case History*. Nia is a 51-year-old Black divorced female with two adult children. She worked as a director of a childcare center and holds a graduate degree. She reported a medical history of asthma, arthritis, and eczema. To her knowledge, she never had COVID-19. She received the Pfizer COVID-19 vaccine and boosters as soon as they were available due to workplace vaccine mandates. She was a proponent of traditional childhood vaccines, hesitant about flu shots, and critical of the COVID-19 vaccine.

*Traumatized by COVID-19 vaccine side effects*. At the time of the interview, Nia reported many physical ailments. Given the timing of these symptoms, rushed development of the vaccine, and lack of a medical diagnosis for these ailments, she attributed these symptoms to side effects of the COVID-19 vaccine. As a result of the first COVID-19 shot (of the two-part series) she experienced a headache. After she received the second shot, she experienced a myriad of symptoms, including sneezing “all day” not explained by allergies, coughing that was not attributed to asthma, respiratory issues, and rashes. In her words,

*So when I took the [COVID-19] vaccination, I started having really bad headaches. Mucus was running from my head out my nose so bad, to the point where I was using up whole rows of those Dollar Tree paper towels a day… And this never happened to me in my life. Sneezing all day. I was sneezing all day. No allergies, asthma, not red, nothing*.

She also experienced excruciating nerve pain in her fingers and legs that negatively impacted her functioning, especially at night. As she described,

*And ever since that COVID [vaccine], my fingers, like, my nerves, they jump in my fingers jumping by itself. My leg is jumping because it’s hurting so bad that my nerve is jumping. And it gets so irritable. At night, I can’t even sleep. I have to sometimes take sleeping pills just so I can have a good night rest because I’m up all-night burning, tingling, and my nerves is jumping*.

In addition to these intolerable symptoms, she mentioned recently developing two black patches on her arms that raised alarm because they were unlike rashes due to eczema. Despite seeing numerous specialists and having x-rays, bloodwork, and tests done for hereditary diseases, she had no answers as to the root cause of these symptoms. With a lack of alternative sources, she attributed these to the COVID-19 vaccine.

*Views on traditional childhood and other vaccines*. Despite being opposed to the COVID-19 vaccine, she indicated she was up to date on all early childhood vaccinations and those needed to work in childcare facilities (for example, hepatitis, measles, mumps, COVID-19 first series, and boosters). Her views on traditional vaccines (for example, MMR vaccine) were favorable for herself and her children because she knew the importance of being protected from childhood diseases, and she believed the protection was long term. In fact, she expressed valuing some vaccine mandates because without the protection she may have died:

*Now if [it] was something mandatory like yellow fever, malaria, chicken pox, measles because I almost died from chicken pox. I’m glad I had the vaccination because then I didn’t have it. It probably would have killed me because I was 33 when I caught that and I had a fever of like 110 and would have to be incubated in the hospital so they got my body temperature down*.

When asked if COVID-19 influenced her views on getting vaccinated for the flu, she said:

*Actually, with the flu shot, I was getting it a long time ago. I used to be really a fan of it. Every year they were giving me the one—I think it was at CVS—they would give me the one in my nose… I never had the flu. I never caught the flu because I drink a lot of orange juice and take vitamin C tablets and I take iron, so I really don’t do the flu vaccination*.

*Distrust Invoked by Mandates and Misleading Communication*. When asked about her views on the COVID-19 vaccine, she stated: “I feel like we [childcare providers] were forced to take it. I really didn’t want to take it because I know my body and things don’t take well with me. So, I was kind of scared.” She also said, “Yeah, a lot of people, we didn’t have a choice. If you don’t get it, you won’t have a job.” She believed the rollout of the vaccine was so rushed that no time was allotted to learn more about it to make an informed decision. As she had suspected, the persistent symptoms she experienced after the COVID-19 vaccine reinforced her fear of receiving a future COVID-19 vaccine or other vaccines rushed in development. She said,

*I didn’t even have a chance to read on the stuff to make sure that it didn’t have something in [it] that I could have been allergic to. Like you guys didn’t even give us a chance to read or make a decision on our own. We was forced to do that. Because if I hadn’t known this is what I’m going through now, I would have never took that stuff*.

Exacerbating her distrust of the COVID-19 vaccine was the inconsistency in vaccine messaging during the pandemic by public health officials and on the news. Nia reported feeling tricked by pandemic communications:

*Yeah, what I mean by being tricked is “if we give you this, it’s going to protect you from COVID.” Then after we take the vaccine, in 2 days later on the news, there’s no guarantee that you might not catch COVID. That’s how I feel like we were being tricked*.

Of the available brands, Nia selected the Pfizer COVID-19 vaccine on the basis of the company’s established history and talking directly with nurses at Pfizer. Despite being vaccinated, she believed the COVID-19 vaccine was overrated because it did not provide the long-term protection offered by vaccines for other infectious diseases such as hepatitis. The evolving strain of COVID-19 meant that more than one series of shots was needed. To her, an effective vaccine should not require repeated administrations such as boosters annually or for new strains. She stated, “This stuff you get only lasted 6 months and we’re back to square one.” She expressed skepticism related to pharmaceutical companies whom she claimed were not getting the “right ingredients” because it did not work long term, which caused her to believe she was being “tricked.” She distrusted communications that leveraged power figures taking the vaccine as evidence that the COVID-19 vaccine was safe. She viewed this messaging approach as follows:

*Because you’re saying it’s safe and even when Trump took it, who’s to say he took the vaccine? That could have been the placebo. I don’t know, but they given it to us to convince us, “Oh, the president took it, you should take it.”*

Nia described that she currently took a holistic approach to disease prevention. She mentioned how she takes orange juice, vitamin C tablets, and iron to prevent the flu rather than getting the flu vaccine. She remarked that medicines were created to sustain big corporate companies when there are other, more naturalistic approaches that “can cure the human body’s needs and deficiencies.” She indicated she would not take a future vaccine until there was a significant body of research on its safety and effectiveness. At the time of the interview, she preferred to promote her health with natural remedies.

*Need for informed consent*. Another element of distrust emanated from conflicting information from public health officials such as Dr. Fauci about COVID-19 and the safety of the vaccine. When asked what she would recommend public health experts avoid doing or saying in future pandemics, she urged public health officials to verify information before they share it with the public. She said,

*I think they should have 100% researchable information and they should be educated on what it is. They’re telling us 100% before they put it out there, because this one was really bad every day… every day they was coming out with conflicting information. This one is saying one thing, then they saying, “Oh, somebody told us that you don’t have to be afraid.” Then they come back and say something else, like, get all your facts straight before you come and put this platform into view for the public, the whole world to see. Let us know the research that you’re doing. Make sure that it’s all set in stone so nobody will have to go back years later to reinvestigate what you said and find out that it wasn’t even the truth*.

When asked what public health officials should do in the future to reach people like her and her family, she highlighted a need for health professionals to ensure patients are informed when providing consent to be vaccinated. She mentioned that her parents were illiterate and suggested health officials should sit down with patients “Zooms [format of the interview] like this or clinics” to explain vaccines step by step and check that they understand before making a decision.

*Stress, Discrimination, and Social Supports*. Nia indicated experiencing several thorns during the pandemic. She felt there was a lot of governmental corruption that was covered up during the pandemic. She said the paranoia from the pandemic caused a lot of racial tension. She said “People was walking up hitting people in New York. Some of my friends are Asian; they got taunted really bad at where they work at.” Further, she noted that people distrusted each other and were so fearful that they were going to spread the virus and infect them that they stopped interacting with each other. She was concerned about childcare providers running out of money, despite being financially stable herself. Work as a childcare provider was incredibly stressful for her during the pandemic, so she and her co-workers relied on each other to mitigate the stress when they were not attending to children: “we just found a way to just everybody go out and take time to go in the kitchen and make them a sub sandwich that we brought a—we had a catfish fry. We’ve just been doing things to just keep us grounded…”.

*Dual views on vaccines*. Nia is someone who expresses duality in her views on vaccination in general and COVID-19 in particular. Unlike other vaccines (for example, traditional childhood vaccines), she did not acknowledge that the COVID-19 vaccine may have prevented her from contracting COVID-19 and developing a more severe illness. She also did not mention the importance of getting vaccinated to protect the children in her care.

Overall, she supports vaccines and believes they have protected her against serious childhood diseases. She emphasizes knowledge and research are important when making decisions about vaccination. Her distrust of the COVID-19 vaccine is predicated on (1) her unexplained symptoms that coincided with receiving the second COVID-19 vaccine dose, (2) the hurried development of the COVID-19 vaccine, (3) being “tricked” into thinking the COVID-19 vaccine would provide full/long-term protection, (4) misleading and unsubstantiated messages about vaccine safety and efficacy, and (5) being forced to vaccinate as a condition of employment.

### Cross-case theme summary

Collectively, these three cases span the continuum of vaccine hesitancy and uptake from distrustful and resistant to accepting and adopting and in between with resentment for mandated quickly developed vaccines but acceptance and uptake of long-standing, tried-and-true preventive vaccines. These cases have several similarities and differences across ten key findings or “themes” summarized in Table 1. These include: vaccine attitudes; decision-making motivations; prioritizing family member health; the role past vaccination trauma plays in decision-making; systems of social support significance; the importance of information to guide decisions; (dis)trust in news, social media, and politicians; disappointment in humanity; future recommendations including respecting individual autonomy; and openness to future vaccines.

**Table 1:** Summary of cross-case themes

	Case 1: Alexis	Case 2: David	Case 3: Nia
Vaccine attitudes	Opposed to all vaccines (except did vaccinate once to attend a trip)	Accepting of all that have been tested	Accepting of traditional childhood vaccines
Trauma with past vaccines	Son’s negative reaction to MMR; had son vaccinated with slower schedule of vaccines before age 3; self and son had negative flu experience; extended family members experienced negative responses to vaccines	No past trauma; did choose to have a slower schedule for child vaccines for youngest child who was at higher risk due to preexisting health conditions	Experienced negative health outcomes from COVID-19 vaccine; past negative reaction to chicken pox resulting in favorable attitudes for that vaccine; receives flu vial nasal administration (not shot)
Care for family members’ health	Not want son to experience negative response to a vaccine (prevent what happened in the past); home schooled son to reduce his exposure	Made cautious decisions to keep youngest child safe; homeschooling long after others returned to school	Espoused childhood vaccines to keep children safe; concern that COVID-19 vaccine was unsafe for people
Social support significance	Recommitted to and sought out social support networks such as church and love tribe and going on scout hiking trip	Avoided going out as a family; has a neighbor family to interact with (harder for wife to not have social connection)	Turned to co-workers during the day when children slept to decompress from the stress of the pandemic
Decision-making motivations	Decisions about vaccination heavily influenced by past traumas and need to keep son safe. High premium on respecting that people know what is right and comfortable for them. Believes in science to a certain degree	When making decisions, was highly motivated to keep family safe and keep others safe (for community good). He shared information with others in digestible ways (to promote vaccine) during the pandemic. Believes in science strongly	Decisions influenced by past side effects experienced by illness (for example, sick with chicken pox, so motivated to take vaccine), employment pressure, length of vaccine development/ research, and vaccine side effects compared with long-term benefits
Value information	High: get information from non-governmental sources, old textbooks	High: get information from government and scientific literature	High: sought out information from pharmaceutical vaccine developers
Trust in social media, news, politicians, and medical community	Major distrust of social media, news, politicians, and medical professionals. Believes that big pharmaceutical companies influence the content of news, media and politicians via funding them	Trusted CDC and other reputable governmental sources of information. Recognized the disconnect of what was put forth in the media and what he was privy to when he was making decisions	Believed she was tricked by pandemic messaging; went to big pharmaceutical websites for information; did not trust government sources; believes big pharmaceutical companies values making money when natural remedies work
Disappointment in humanity	Disappointed that people do not recognize that others need to make choices for themselves and context	Observed that people struggle to make logical decisions using scientific information	Appalled by racism against Asians and paranoia of contracting COVID-19 making people fearful of each other
Future recommendations	Make sure information is provided so that people can provide truly informed consent	Presenting objective information to others so they are free to use it to make (hopefully) informed decisions. People hate being told what to do or feeling their choices are being taken away	Make sure information is provided so that people can provide truly informed consent
Future vaccines	Will not say “never” but will not agree to vaccines based on mRNA	Willing to uptake future vaccines after reviewing data	With enough research and few side effects, would consider future vaccines

### Themes

All three participants care deeply about the safety and well-being of their families. They seek out and digest information to the best of their abilities with the goal of protecting themselves and their family. Their preferred sources for information and trust in authority varied, with Alexis having the most skepticism toward mainstream media and the government, Nia somewhat less so as she did look for information on Big Pharma websites (for example, Pfizer) but also voiced extreme distrust of Big Pharma and the government. David had full trust in government-released information and had access to the same primary sources of information used by government officials to make recommendations. However, all three emphasized the importance of providing people with the information necessary to make informed decisions around vaccines and health.

Alexis, David, and Nia had different underlying beliefs that guided their health-related decision-making. Alexis was influenced much more by spiritual messaging and explanations than by science or data and opposed vaccination in general. Nia’s distrust of Big Pharma and the government led her to prefer natural or holistic remedies for sickness, although she still found value in some aspects of traditional medicine (for example, childhood vaccines). David used only data and science to guide his understanding and decision-making around health decisions and fully participated in Western medicine practices and was an advocate for vaccination. Trauma with past vaccine side effects also greatly influenced both Alexis’ and Nia’s decision-making and hesitancy around vaccination, currently and for future vaccines. David did not have a history of negative vaccine side effects and was willing to consider future vaccines after reviewing relevant data on safety and efficacy.

Social support was an important factor in all three participants’ well-being. Alexis got her support from her son, church, and her spiritual “love tribe.” David was closest to his immediate family and relied on them for almost all of his support, and Nia had social connections with her co-workers and daycare parents. Having others with similar worldviews to experience and process the pandemic with was a significant support for all three participants.

All three participants also valued autonomy in decision-making. They voiced the opinion that one size does not fit all and that compliance is more likely when individuals can use information to make their own decisions. Alexis and Nia also felt that having as much information as possible was critical to obtaining truly informed consent. All three participants voiced a level of disappointment in humanity, related to a lack of respect for individual choice (Alexis), an inability for people to use scientific data to make logical decisions (David), and the us versus them mentality that grew from contentious differences of opinions during COVID (Nia).

## Discussion

Vaccine uptake is an essential component for managing epidemics and pandemics. During the COVID-19 pandemic there was intense national dialogue around vaccines. Many factors influencing intentions to become vaccinated or not centered around demographic factors, risk and benefit calculations, social factors, and beliefs about vaccines. Through their voices, the three cases presented here capture contextual and attitudinal influences and a more nuanced and multifaceted nature of decision-making about whether or not to become vaccinated against a novel infection with new vaccine. By drawing on rich, nuanced information, these three case studies offer unique and novel insights into how the dialogue around vaccine uptake should evolve to meet the needs of different people. These cases demonstrate that we cannot approach vaccine campaigns using a one-size-fits-most messaging strategy that treats non-adherers as a homogeneous adversary group. People who remain unvaccinated are motivated by different rewards, fears, values, and barriers that undergird their personal risk–reward calculations and drive their behavior. Individual behaviors are motivated by personalized risk–reward assessments on the basis of perceptions influenced by information and values [16]. For example, Alexis remained unvaccinated due in large part to previous adverse vaccine reactions and having medical knowledge, training, and professional experiences that supported her views on and distrust of vaccination. As a risk assessor, David had access to information not available to the general public, which, along with his favorable views on the power of vaccines for preventing worse outcomes, guided his decision to get vaccinated. Nia was generally in favor of vaccines, but became suspicious of the COVID-19 vaccine due to its rushed development, adverse side effects she experienced, and belief that she was forced to be vaccinated as a condition of employment.

Far too often, public health messaging campaigns assume a rational actor model: tell people vaccination is helpful and they will start doing it; if not, they are labeled as “irrational.” This judgment stems from misguided assumptions that humans make decisions linearly with the same value structure and risk–reward calculation. Thus, precision prevention via messaging tailored to risk–reward calculations and individual and community-level social determinants of health (for example, reach and access barriers such as distrust, fear, discrimination, economics, structural constraints, and healthcare and education access) will be essential to improving vaccine acceptance and uptake. Messaging misaligned with preferences, values, risk–reward ratio, and social determinants of health likely results in detrimental outcomes by eliciting reactance or non-adherence [17]. These undergirding factors must be understood to inform tailored messaging responsive to needs and psychological dispositions [18–21].

This multiple case study has several strengths worth noting. The three cases described in this report represent varying decision-making processes and views on traditional and COVID-19 vaccines. The data richness of these cases provides insights into similarities and differences across the lived experiences of three people that span continua of distrust, trauma, doubt, and prevention reactions to the COVID-19 pandemic. Some spoke of mandated vaccination and how it impacted their beliefs about current and future vaccines. Unlike other qualitative studies published on COVID-19, these interviews not only captured views on vaccines and decision points, but also addressed recommendations for future pandemics.

Although the three cases provide varied vaccination decision-making processes, there are several limitations worth mentioning. By their nature, case studies are not generalizable. We recognize our three cases are selective. The three participants had bachelor’s degrees or higher levels of education. They sought out information to inform their decisions, albeit their sources varied from conservative podcasts to government websites to pharmaceutical company websites. All three cases were middle-aged adults with children (either minors or fully grown), thus their risk factors and pressures to follow public health guidelines may differ from younger and older adults.

## Conclusion

Understanding, accepting, and empathizing with how people’s lived experiences with past vaccines influences their decision-making regarding present and future vaccine acceptance and uptake is vital to inform current and future public health approaches. One size does not fit all. People want to be heard and not dismissed. Being treated as irrational, “crazy,” or different leads to feelings of resentment and hurt that are harmful beyond the medical concern de jour [for example, COVID-19 vaccine yesterday and today leads to concerns over Lyme vaccine tomorrow and human immunodeficiency virus (HIV) vaccine in years to come]. As shown across these case studies, there are legitimate skepticisms people hold about vaccines in particular and medicine and public health in general. By honoring past traumas and sources of distrust of prospective and current clients and patients, public health and medical professionals can begin to engage the hesitant and resistant in meaningful conversations that speak to clients/and patients’ values and concerns, meeting them where they are in their thinking and preferences, and providing them the most credible and relevant information and tailored communication so they can make the best decisions for themselves and their families, even if those decisions are contrary to the recommendation these professionals would prefer. This approach will go a long way in building and rebuilding the trust needed for people to listen to and engage with the public health and medical communities.

## Data Availability

To maintain confidentiality and privacy of the case study participant, data will remain protected. Additional coding information, however, can be made available from the corresponding author upon reasonable request.

## Abbreviations

ACIP: Advisory Committee on Immunization Practices
CDC: Centers for Disease Control and Prevention
COPD: Chronic obstructive pulmonary disease
COVID-19: Coronavirus disease 2019
DNA: Deoxyribonucleic acid
ER: Emergency room
MERS: Middle East respiratory syndrome
FEMA: Federal Emergency Management Agency
HIV: Human immunodeficiency virus
HPV: Human papillomavirus
MMR: Measles, mumps, and rubella
mRNA: Messenger RNA
RNA: Ribonucleic acid
SARS: Severe acute respiratory syndrome
TD: Tetanus and diphtheria
UIUC: University of Illinois at Urbana-Champaign
